# The Map’s Design: Evolution’s Impact on Navigation and Spatial Cognition

**DOI:** 10.3390/ani15243628

**Published:** 2025-12-17

**Authors:** Isabella S. Olynik-McLaughlin, Diano F. Marrone

**Affiliations:** Department of Psychology, Wilfrid Laurier University, Waterloo, ON N2L 3C5, Canada; olyn5490@mylaurier.ca

**Keywords:** cognitive map, hippocampus, neuroethology

## Abstract

Spatial cognition is fundamental to an animal’s ability to move through and interact with its environment. Thus, understanding the physiological basis for this cognitive function and how it varies across taxa is fundamental to understanding how ecology alters brain architecture and function. Although elements of spatial cognition can be found across the animal kingdom, here we concentrate specifically on cognitive maps and their physiological basis. This will begin with a focus on the rodent model, which is the source of the vast majority of data on the neural basis of cognitive maps. This serves as a foundation for comparative analysis of other taxa to demonstrate that, although this line of inquiry is relatively new and small, important variations have been observed, tied largely to changes in both perception and ambulation, that may fundamentally alter how spatial cognition is implemented. This latter analysis emphasizes birds, as recent data in this order provide fundamental insights into how the selective pressures that drive changes in peripheral physiology also drive commensurate neuronal changes in spatial cognition.

## Simple Summary

Understanding and navigating through space is important to survival, with brain systems having evolved to support this function. Over the past several decades, we have built a comprehensive understanding of these systems, but this knowledge comes from a limited number of model species, most notably rodents. As a result, we risk missing important ways that these systems vary across the animal kingdom. Recent data show that evolutionary changes, notably in perception and motion, change the way animals think about space. These data reveal insights into how the evolution of the body has also driven changes in the brain.

## 1. Introduction

For mobile animals, efficient navigation is a key component of survival. Navigation permits both approach to rewards and escape from threats. Given the potential benefits to be reaped from better understanding space, it is perhaps not surprising that elements of spatial cognition can be found across the animal kingdom. Upon this foundation, however, evolution has sculpted additional cognitive abilities to enable progressively more complex strategies suited to varying ecological niches, culminating in the cognitive map. Understanding how cognitive maps are implemented physiologically and (importantly) how this implementation varies across taxa promises to deepen our understanding both of the basis for spatial cognition in general and of how it is shaped by pressures in the natural environment.

Decades of research have been conducted on this topic and have built a relatively comprehensive model for how spatial cognition supports navigation and memory. Here we first describe many of the general themes that have been uncovered to date in our understanding of spatial cognition, focusing on the rodent model. This provides a foundation for comparing how variations in these basic principles shape the implementation of spatial cognition across taxa. This latter analysis will focus on recent data, particularly from birds, that have fundamental implications for our understanding of how evolution sculpts the neural architecture underlying spatial cognition. Before focusing on cognitive maps, however, it is worth providing some context on the maps relative to other strategies of navigation, such as path integration.

## 2. Foundations of Spatial Learning—From Path Integration to Cognitive Maps

At its simplest level, in theory, nothing is required for spatial cognition beyond perception and simple conditioning. Instrumental conditioning allows stimuli to become associated with outcomes, and animals can thus learn to move toward stimuli associated with rewards and away from stimuli associated with punishments. A squirrel seeking a fallen apple could, in theory, find its target simply by detecting the appearance or scent of an apple tree and wandering at random until it encounters one, much like organisms that rely on chemotaxis or phototaxis. It would also be possible to build a route upon this kind of beacon following by navigating to other landmarks that, through conditioning, have become associated with moving progressively closer to the tree (e.g., brick house → tin shed → wooden fence → apple tree). It is immediately apparent, however, that this most minimal form of navigation is incredibly inefficient. One of its biggest flaws is that, in isolation, this information provides no flexible means to return to this tree if the animal must leave via a novel route or if conditions change. Under such circumstances, every reward is largely found based on chance alone.

To solve this problem, more information needs to be acquired and associated with this tree. The simplest addition would be some sense of heading (either from an internal compass or external cues) and a sense of travel (either by detecting the distance traveled or by a sense of time). These two pieces of information permit path integration—that is, the ability to construct and add to vectors over the course of navigation [1]. With this information, our squirrel’s ability to return to the tree is limited only by the accuracy of these senses and its capacity for adding these vectors. Path integration was first postulated by Charles Darwin [2] and has been intensely studied since, primarily in the context of homing behavior. In such cases, an animal explores the environment and, upon finding food, immediately returns home in order to share it with a group, feed young, or add to a hoard. One notable example is *Cataglyphis* ants, which take direct-line return paths to their nests following long, tortuous excursions of over 1000 m across featureless saltpans (e.g., [3,4]).

Although not as grand as the *Cataglyphis* ants, many examples of path integration can be found across a broad range of species (for reviews, see [4,5,6,7]), and thus may be conserved across much of the animal kingdom. As powerful as path integration is as a navigation strategy, it has some notable shortcomings. Consider the following scenario: Our squirrel encounters a predator and must flee. Its escape path becomes tortuous as it darts and weaves to avoid capture, making it impossible to track heading accurately. How, then, can the apple tree be found effectively under such conditions? Animals solve this problem using a mental analog of a topographical map—a “cognitive map” [8].

Edward Tolman [8] described cognitive maps as flexible internal representations of a given environment, into which spatially relevant information can be integrated. By using rats as an animal model, he demonstrated that factors such as salient cues, path repetition, and task difficulty are incorporated into a mental framework. This framework can then be called upon to produce behavioral responses in an environment. Ideally, such a representation will encode the geometric relationships between all salient stimuli. Such a map permits an animal to infer its location on the basis of observed landmarks—what Gallistel [9] refers to as piloting. Piloting thus allows finding the apple tree from any arbitrary location so long as some familiar landmarks are present.

Given the flexibility conferred by cognitive maps, it is perhaps not surprising that there are numerous examples of map-like navigation across the animal kingdom, including humans (reviewed in [10]), non-human primates (reviewed in [11,12]), rodents (reviewed by [13,14]), birds (reviewed in [15,16]) goldfish (e.g., [17,18,19]), and turtles (e.g., [20]). If cognitive maps are widespread across species, a key question to be addressed is how the necessary representations of space supporting these maps are implemented across diverse neuronal architectures.

**Figure 1:**
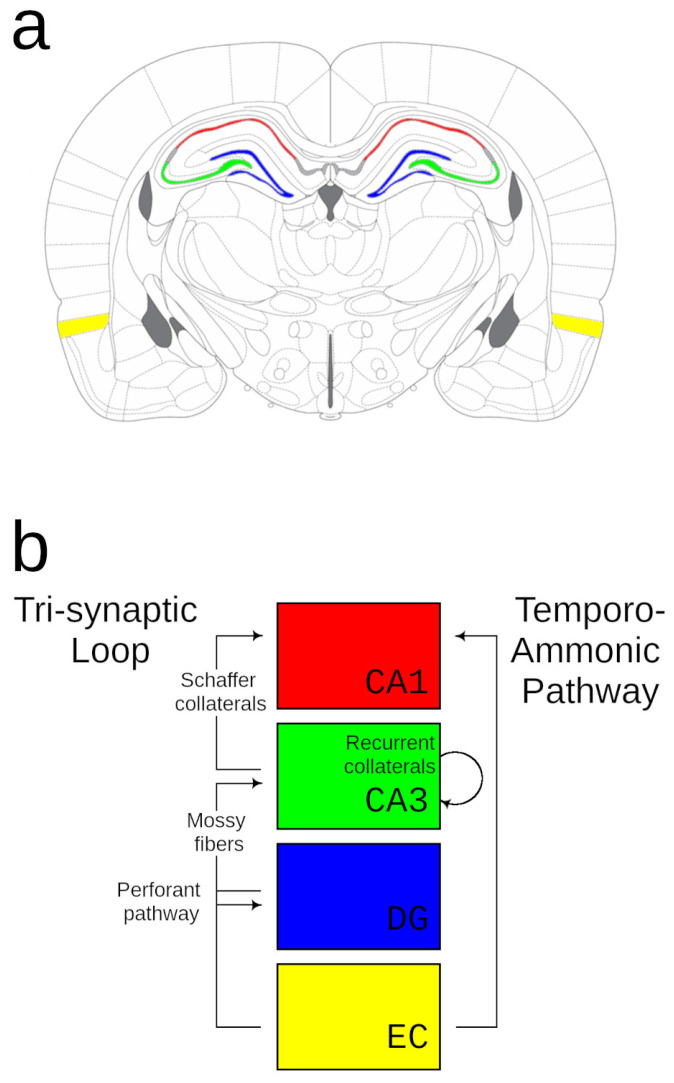
The canonical mammal HF. Above (**a**), a coronal plate (adapted from [21]) shows the major components of the rodent HF, colored to match the corresponding schematic depicted below (**b**).

The rodent as a model organism has generated more data on the physiology of cognitive maps than any other species (see Figure 1, adapted in part from [21]). Accordingly, this review begins with a brief synopsis on the physiology of cognitive maps in these animals before comparing this wealth of knowledge with recent data from other species.

## 3. The Physiology of Cognitive Maps

A fundamental milestone in our understanding of the physiology of cognitive maps comes from the seminal work of O’Keefe and Nadel [22], who argued, primarily on the basis of anatomy and lesion data, that the hippocampal formation (HF) provides a neural system by which cognitive maps are formed. The subsequent decades have seen an explosion of data, largely in rodents, that are broadly consistent with their model of hippocampal function. While it would be impossible to summarize the thousands of papers on this topic in their entirety, here focus is placed on a few key features of the mammalian HF to provide the framework for later comparison.

The fundamental unit upon which cognitive maps are built is the place cell. Throughout the HF, neurons develop spatially selective firing patterns (‘place cells’) and fire high frequency bursts of activity whenever the animal enters a specific location that the cell is responsive to (a ‘place field’) within a given environment [23]. In rodents like rats and mice, place cells are abundant, and a considerable portion of neurons recorded from the hippocampus exhibit fine tuning to a precise position in space. Each cell responds to a slightly different location, and so collectively form a representation of the external environment. These neurons are thought to respond to polymodal sensory information both in the external environment and internal to the animal. Importantly, place cells encode information beyond sensory cues, with their activity modulated by task demands, reward value, and motivational states, in addition to changes in the environment (e.g., [24,25,26,27,28,29,30,31,32,33]). This polymodal integration is part of what makes place cell coding intriguing not only for understanding navigation, but as a fundamental building block for memory in general.

The polymodal integration that makes the HF attractive as a hub for ‘indexing’ memories stems from the fact that this structure receives massive convergent input from across the brain. This very convergence, however, creates unique challenges. A single, homogeneous neural network cannot efficiently process all the facets of memory simultaneously. A more efficient solution is to tackle the vast amount of information through several processing modules that each make unique contributions to memory formation and retrieval. There are a number of reviews dedicated to this topic (e.g., [34,35,36,37,38]) and so only those principles important for comparison with recent data from other species are highlighted.

### 3.1. Functionally Segregated Inputs to the Hippocampal Formation

The entorhinal cortex (EC) is regarded as a major communication hub between the neocortex and the HF. Superficial EC layers provide input to the HF, while its deep layers and the subiculum are thought to provide output from the HF to the rest of the cortex. It is important to note here, however, that the information coming in from these cortices is not uniform. Rather, different information is conferred to the HF from different EC subregions. The lateral EC (LEC) responds to discrete stimuli [39] and displays only weak spatial tuning, while neurons within the medial EC (MEC) fire in multiple discrete spots in an environment, giving their activity a grid-like structure [40]. It has been proposed [41] that LEC carries information about external cues and their positions (in egocentric coordinates), whereas MEC carries allocentric information about the organism, including its position. These specialized neurons in the MEC provide a topographical matrix that covers the whole surface of an available environment [42]. These cells become activated in specific vertex locations (‘phases’) that remain stable even when landmarks are removed from the area. The firing patterns of these neurons generate a hexagonal pattern, hence why they are commonly referred to as ‘grid cells’. Evidence suggests that the firing of grid cells provides a metric for navigation in large and complex expanses of space [43]. Importantly, the MEC grid cells may be critical for path integration in mammals [44], as they provide an accurate [45] and efficient [46] code for space that is exquisitely sensitive to self-motion signals [47].

Moreover, input from EC reaches the HF via two distinct pathways—a direct temporoammonic pathway from EC to CA1 and an indirect perforant pathway that projects to the dentate gyrus (DG) and then CA3 before projecting finally to CA1, forming the well-known tri-synaptic loop. Although most research has focused on the tri-synaptic loop, recent data show that the monosynaptic projections from both the LEC and MEC are fundamentally important for hippocampal maps. Destruction of the direct monosynaptic projection [48] abolishes the precise spatial tuning of place fields in CA1, while interruption of CA3 [49,50] or the dentate [51] leaves CA1 place fields intact, although less stable [52], and spares some forms of spatial learning. What, then, is the functional role of the tri-synaptic loop?

Inputs from EC to the dentate gyrus are suggested by many models to decorrelate similar inputs [53,54,55,56], while in CA3 (the next stage in the loop), recurrent connections store novel representations that can later be restored through “pattern completion” even to partial cues [53,54,55,56,57]. In turn, CA1 (the main target of CA3) is thought to provide a comparator function to contrast current sensory input (from EC) with the patterns stored in the CA3 recurrent network and return this information, through the subiculum, to the EC [24,58,59].

### 3.2. Functional Gradients Within the Hippocampal Formation

Early models viewed the hippocampal subregions as uniform units arranged in a largely feed-forward (serial) manner. We now know, however, that inputs from different structures arrive at the HF in several different gradients, both along the long septo-temporal axis (e.g., [60]) and the transverse axis (e.g., [61,62,63]) of the tri-synaptic loop. Both of these gradients have important implications for spatial cognition.

Moving from the septal pole of the hippocampus (often referred to as the dorsal pole in the rodent) towards the temporal (ventral) pole, significant differences can be observed in connectivity [64,65,66] as well as gene expression (e.g., [67,68,69]). These differences are thought to culminate in systematic changes in information processing along this gradient. Near the septal pole, place cells are numerous, with small receptive fields and a high spatial information content. Towards the temporal pole, place cells become less numerous in a single environment, and their receptive fields expand, reaching the scale of meters, rather than centimeters [70,71,72]. Moreover, comparable functional heterogeneity along the longitudinal axis can be found in a number of other species, including humans (e.g., [73]), non-human primates (e.g., [74]) and multiple species of birds (e.g., [75,76])

Although septo-temporal gradients impact HF function in a number of ways, the most relevant observation here is in the functional diversity of CA1. This is because in the tri-synaptic loop, axons from the spatially tuned MEC and non-spatial LEC converge on the same population of cells in the dentate and CA3, enabling integration of both types of information. This integrated information is in turn passed to CA1 uniformly via Schaffer collaterals. In contrast, the direct temporo-ammonic projection preserves the gradient of the EC. That is, MEC projects preferentially to the proximal (near CA3) region of CA1, while LEC projects primarily to distal (near the subiculum) CA1 [77,78,79].

The only study to explicitly search for a medio-lateral gradient in place cell dynamics in birds [76] found no evidence for such organization. This observation must be interpreted with caution, however, because these functional gradients are dynamic (at least in rodents): by coupling oscillations, cells can be ‘tuned’ to increasing the drive from some inputs while dampening others [80,81], and so these gradients need to be studied under a variety of behavioral conditions.

Collectively, these data provide a detailed model of how spatial maps are constructed in the rodent hippocampal formation—a framework that, in many respects, generalizes across species. However, there are likely limits to how far the rodent model can be applied to spatial cognition more broadly. There may be limits, however, to the application of the rodent to problems of spatial cognition across species. Rodents, like many other animals, are exquisitely adapted for a specific ecological niche—for a nocturnal, olfactory-driven and largely tunnel-dwelling existence. As we extrapolate data derived from these animals to other species with fundamental differences in the way they both perceive information from and act upon the world around them, we might expect this to also create fundamental differences in the way space is computed within their brains.

## 4. Ecological Demands Shape Biological Differences

When comparing spatial cognition between species, it is important to consider the ecological niche to which each species has adapted. As per Gibson [82], an ecological niche is a collection of affordances, which he characterized as a set of abilities that an animal can possess within the confines or expectations of a given environment. Thus, the repertoire of the animal is a fundamental, inseparable property of the cognitive map. This notion is supported by many observations that the actions of an animal form a key part of the spatial coding within the HF (e.g., [83,84]). Place field size (and thus resolution) is tightly coupled to movement speed [85,86,87]. Further studies have ruled out optic flow and vestibular cues as the source for this coupling and point to an efference copy (an internal signal of self-generated movement) converging on the hippocampus as a likely mediator of hippocampal place coding [88]. This notion is consistent with both early reports that hippocampal theta peaks immediately precede motor responses [89] as well as a recent study [90] attempting to disambiguate movement trajectories from other variables (e.g., external cues, context, rewards) that found that firing of hippocampal neurons was driven by momentary action patterns that transcended all other factors. Thus, motion seems to be a fundamental part of the space coding of the HF.

It is important to note, however, that self-motion is not required for accurate place coding, as animals that are passively moved through space still show normal place cells [86,87]. This coding, however, disappears under restraint [87], suggesting that the hippocampus is encoding possible movement—a state space simulating possible action plans. Such a role fits the importance many have placed on prediction of possible outcomes as a key role for the HF (e.g., [91,92]). In this way, the cognitive map represents a calculation of possible affordances—that is, the relationships between environmental features, their potential outcomes, and the behaviors required to obtain or avoid them. It also inherently reflects the nature of the animal itself—the particular ways it can perceive, move through, and act upon its ecological niche. Each of these facets of the interaction between animal and environment is considered below.

### 4.1. Perceptual Demands

Determining the salience of perceptual cues is critical for understanding how an animal interacts with its environment. Rats, for instance, depend heavily on olfaction as their primary perceptual input. This is in stark contrast to many diurnal animals (including humans) for which vision is the primary sensory driver of behavior. It is intuitive that these differences would sculpt place representations in dramatic ways. Recent observations are consistent with this notion. Outlining these differences, however, requires a brief description of non-local representations.

Place cells are typically defined by the location of the animal—if a cell fires consistently when an animal is in the northeast corner of a maze, that cell is by definition a place cell with a place field corresponding to that corner. However, once the firing patterns of numerous cells are established, these can be used as templates to determine if a neuronal population fires in a pattern consistent with the northeast corner when the animal is actually in a different location—a non-local representation. Experiments doing this have consistently shown that rats demonstrate non-local representations at key points, such as when making decisions at a crossroads in mazes (reviewed in [93,94]). This is an important observation, as one of the integral functions of cognitive maps is thought to be the generation of simulations [95,96]—mental explorations in which an animal imagines a possible outcome as a result of moving to a particular location or engaging in a specific action. A necessary precondition for this sort of simulation is the ability to generate a non-local representation.

Although data from rodents demonstrates that animals can generate non-local representations, a distinct form of non-local representation is seen in non-human primates [97] and some birds [98], termed view cells. A neuron is said to be acting as a view cell if it fires in a similar pattern if the animal is looking towards a specific location or landmark (e.g., the northeast corner of the maze), regardless of the animal’s actual location or orientation. This sort of activity has not been reported in rodents; rather, it is head direction cells that have been extensively described in these animals. Unlike view cells, head direction cells tune to a preferred direction based on the orientation of the animal’s head independent of location, all of which rotate uniformly in response to the shifting of a salient environmental cue [99].

The presence of non-local representations across such diverse taxa underscores the shared computational principles that underlie cognitive maps. These maps, which arise directly or indirectly from sensory inputs distributed throughout the brain, constitute some of the most polymodal and information-rich representations possible. It is therefore reasonable to assume that they are robust to differences in the precise mix of sensory modalities across species.

Why, then, do rodents appear to lack view cells? One possibility is that rodents’ relatively poor visual acuity, panoramic visual field, and lack of a fovea [100] make them less dependent on visually anchored representations. Nevertheless, rats clearly use distal cues to aid navigation: manipulations of these cues impair performance on many spatial tasks (e.g., [101]) and degrade hippocampal place cell representations (e.g., [84]). Thus, while rodents can form non-local representations, the specific form of view-based coding seen in primates and birds may not emerge in a species whose spatial cognition relies less on visual detail.

The presence of view cells may in fact be a product of a bias in navigational strategy, rather than in the nature of information processing within cognitive maps per se [102]. For an animal like a bird or a primate that has excellent vision, distant visual landmarks provide cues that are both reliable and relatively safe (since they can be sampled from far away) for updating position information in real-time. This notion is consistent with the observation that place coding in primates is almost exclusively non-local under some conditions ([103,104], but see [105]). Commensurately, it remains possible that non-foveal animals possess view cells but rarely integrate them into spatial maps, if ever, due to an ecologically driven bias to prioritize cues such as odor and somatosensation that have an inherently lower range.

Another possibility is that rats do, in fact, demonstrate view cells, but we have yet to observe them simply because the lack of fovea makes it incredibly difficult to establish the direction of gaze in rodents. It is worth noting that a recent paper on non-local representations in rodents shows that they sweep outward (perhaps in a manner akin to saccades?) at lateral angles from the head of the rat, consistent with them being driven (or at least influenced) by gaze [106]. If this is the case, they are dramatically lower range and lack fixation, but these differences could plausibly be the product of both the physiology and location of the rodent eye.

Finally, it is worth noting that the hypothesis that non-foveal animals cannot generate view cells suggests that there is a minimal threshold in sensory acuity required for that sense to provide sufficient information to the HF to drive a fixed non-local representation. For instance, if presented with a task in which knowing which response to engage in required pinpointing the origin of a sound, we might expect different non-local representations in the HF of the animal depending on its auditory acuity. Animals proficient in sound localization should be able to conjure “view cell-like” fixed representations of the exact point of origin, while a less proficient animal may provide less detailed representations, perhaps resembling the outward sweeps seen in foraging rats. It should be possible to conceive of a comparable experiment for any sensation, such as an aquatic animal with or without a lateral line to the origin point of a splash in the water. Testing this conjecture across species and with varying sensory acuity may help elucidate the link between variation in perception and variation in spatial cognition.

### 4.2. Freedom of Motion

**Figure 2:**
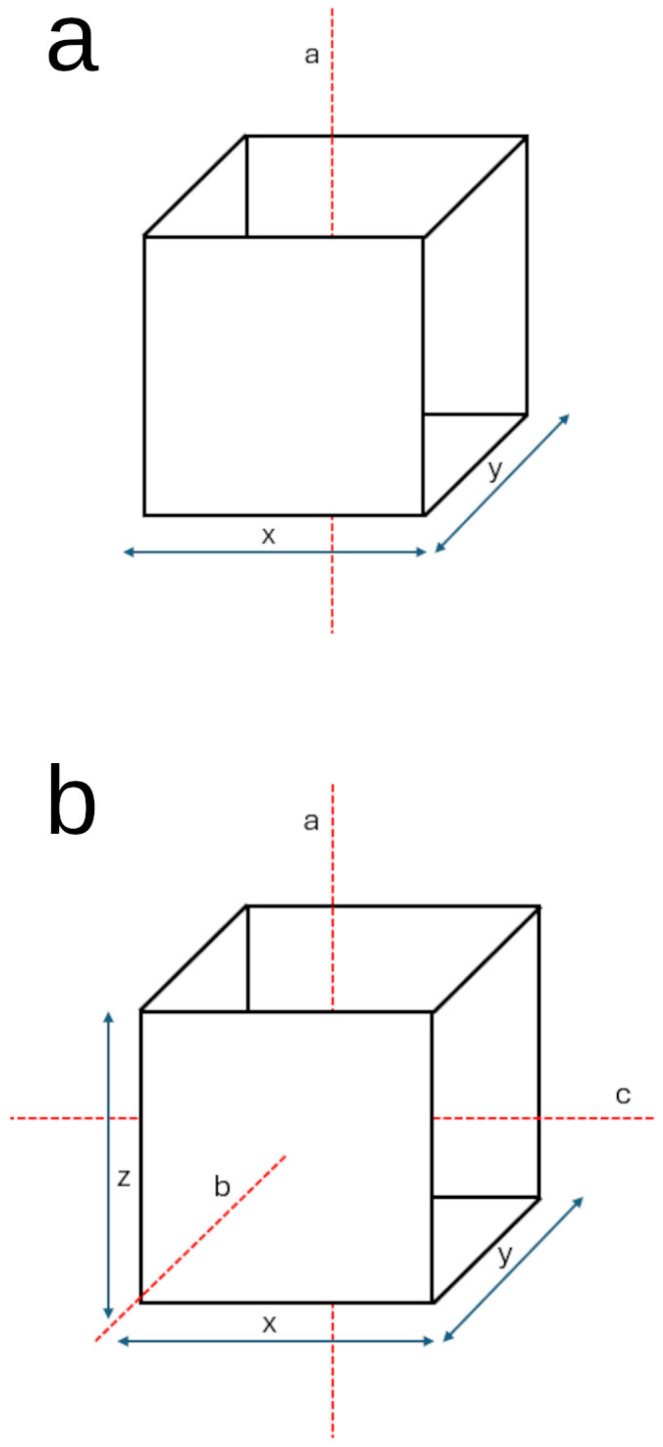
Translational and rotational directions of movement. The diagram above, (**a**), depicts three degrees of freedom of motion, while the lower diagram (**b**) depicts six degrees of freedom of motion (x: left/right, y: forwards/backwards, z: up/down, a: yaw, b: roll, c: pitch).

Another key determinant of how an animal interacts with and processes its environment is the way in which it is able to physically move through it. Freedom of motion is relevant to spatial cognition, as it is a limiting factor of the amount of space an animal can experience both horizontally and vertically. Rather than methods of locomotion, the aspect of motion that is likely to be critical to spatial cognition is the degrees of freedom that any given animal has (see Figure 2). Animals that are restricted to surface ambulation have three degrees of freedom: left/right, forwards/backwards, and yaw [107]. Contrarily, species that possess the ability to travel along the vertical axis have six degrees of freedom: left/right, forwards/backwards, up/down, pitch, roll, and yaw [108]. These classifications are somewhat broad in that they do not distinguish specific modes of ambulation; rather, this system relies more so on the ease with which animals orient themselves along the planes and axes of motion. Thus, one might expect aquatic animals to represent space more like a flying animal than a land-bound one. Bearing this in mind, one might infer that animals that are constrained largely to locomotion on a plane encode space differently than those that move freely through volumetric space.

In a study examining the capacity for land-dwelling animals (like rats) to represent space along the vertical plane, several have observed anisotropy—that is, a higher resolution in the horizontal plane of motion than in the vertical dimension [109,110,111]. In experiments by Grieves et al. [110,111], rats navigated a cubic lattice apparatus that could be reoriented to modify the plane of locomotion. By doing so, the authors could disentangle the influence of motion direction from visual (e.g., horizon) and vestibular (e.g., gravity) cues. When the maze rotated such that one axis was easier to move down, that axis was represented in higher resolution. These observations fit both the notion that affordances shape spatial representations and that the hippocampus represents a repertoire of possible motions to aid simulations of future events (and likely updating this list in real time). Similar findings have been shown in bats, as spatial representations differ when crawling along a surface than during flight [112,113,114].

Taken together, these data clearly show that locomotive affordances shape spatial representation in the brain. However, the extent to which a brain that normally represents space anisotropically is capable of generating isotropic representations under conditions of 3-dimensional freedom of motion (such as during swimming) remains an open question.

This is an interesting question given recent data suggesting that spatial encoding may be isotropic in fish [115]. Banded tetras were trained to navigate towards a goal location in a transparent, rotated Y-maze, in which they needed to go forwards out of the starting box and either continue up and to the left or down and to the right. For testing, the fish were placed into the training aquarium in the starting box without the Y-maze tubes and expected to swim to the same target location within the enclosure. The fish were found to swim closely to their trained trajectory even when unrestricted by the maze, indicating the ability for route replication in a volumetric environment. Moreover, the fish were also trained to navigate through the Y-maze in conditions where either vertical or horizontal information was absent. Researchers noted that the tetras preferred to integrate both horizontal and vertical components of a route when given the opportunity rather than moving along a single plane, unlike rodents [111]. Although no neural data are yet available for these fish studies, the observation that spatial resolution in rats matches their locomotion bias has led to two suggestions [116]: (1) that behavioral biases may serve as a proxy for underlying spatial representations, and (2) that in species such as fish, whose movement is more isotropic, spatial representations may likewise be isotropic.

If this relationship holds true, assessments of the spatial processing of a number of animals become far more feasible. For instance, multiple behavioral studies involving hummingbirds have shown that these birds were more accurate when recalling targets along the vertical than along the horizontal plane ([117,118], but see [119]). These findings suggest that with all things equal, mental representations of vertical space may be better resolved due to internal perceptions of gravity, which might be more acute in animals that move unassisted along the vertical axis. It is not clear, however, how the ability to hover (which carries its own affordances) impacts these observations. Nevertheless, species differences in spatial discrimination may prove invaluable for identifying the most informative model organisms for physiological study. These data reveal that the size and density of place fields are dynamically coupled to affordances. These changes in density have further implications for memory capacity, discussed below.

### 4.3. Density and Information Content of Cognitive Maps

The density (often referred to in the inverse, sparsity) of representations in the hippocampus refers to how many neurons are engaged in the creation of an engram for an episode of experience. This has important functional implications for cognitive maps, particularly in storage capacity. All else being equal, a network can hold more unique patterns if those patterns are more sparse, at the expense of information content per pattern. This trade-off leads to the question of whether different species have evolved different means of negotiating these conflicting demands. Although observations of place cell density in freely moving animals can vary dramatically depending on a wide variety of factors, such as the methods used to sample neuronal activity and the task demands placed on the animal [120], it is clear that reliable species-related differences in the sparsity of population codes exist. A series of studies using a common methodology (open field foraging) and a common method for sampling place cell activity (immediate-early gene expression) may provide valuable insight here. Immediate-early genes have a relatively long history of being used to capture estimates of place cell activity (e.g., [121]). These experiments typically quantify the proportion of cells in the rat HF engaging in transcription during foraging in an open field and yield an estimate ranging from 1/3 to 1/2 of all cells in dorsal CA1 (for instance) expressing place fields in a given environment (e.g., [72,121,122,123]). In rhesus macaques, however, this number decreases to about 5% [123]. While these proportions are radically different, when comparing absolute numbers of cells activated, the numbers observed in macaques were within the range of observations in rats, suggesting (a) there is a minimum number of neurons required to generate robust memory, and (b) as brains grow across evolution, they increase engram capacity rather than the size of individual engrams [123]. Importantly, estimates of comparable foraging-related gene expression in birds suggest this relationship may hold true outside of Mammalia as well. Estimates in both Japanese quail [75] and brown-headed cowbirds [124] estimate an intermediate proportion of active cells in the range of 15–20%. Although precise estimates of the number of cells in these birds have yet to be determined, they are likely much higher than a rodent’s, despite having slightly smaller brains, since birds have much higher cell packing densities [125]. These data fit the notion of engram size being relatively conserved across species and that increases in brain size serve to increase capacity rather than increase information content within individual engrams. Note, however, that these data do not preclude variation within classes. For example, a recent study comparing tufted titmice with zebra finches [76] suggests that variation in the density of place cell representations may exist across Aves. This study showed that, under identical conditions, tufted titmice exhibit approximately one-third higher density of place cells in the rostral-most portion of the HF. The authors know of no comparable comparison across rodents.

## 5. Conclusions

It is clear that the body of literature inspired by the initial observation of place cells has led the development of a deep understanding of the systems driving spatial cognition, thanks in part to many decades of systematic investigation in a small number of model organisms. Given our current understanding, however, the scientific community stands poised to reap the benefits of expanding the investigation of spatial cognition beyond these model organisms to explore the ways in which evolution has sculpted these neural systems alongside changes in peripheral physiology.

Although the existing research that has investigated these questions to date is small, it suggests that changes in the way an animal both **perceives information from** and **acts upon** the environment change the way that environment is represented in the brain, but many more questions remain. For instance, both visual ability and preference fundamentally alter spatial representation, but it remains unexplored if similar changes can be driven by acuity in other modalities. Similarly, the freedom of motion afforded by flight dramatically alters the representation of space, but as of yet, we know nothing about other forms of motion. Behavioral data suggest that movement in water confers an isotropic representation similar to flight (at least in aquatic animals), but we have no direct neuronal recordings. We also do not know the extent to which these differences are driven by experience—that is, does a terrestrial animal have an isotropic representation of space in water, for instance? If not, is this driven by intrinsic physiology or experience? Moreover, the data we have come from animals either restricted to a plane or with complete 3-dimensional freedom of movement. There are also many forms of motion that have freedom of motion in between these extremes (e.g., burrowing or climbing animals, animals with limited flight capabilities) that remain unexplored. In particular, the speed of travel required/possible with varying forms of locomotion may be fundamentally shaping spatial representations.

These data and open questions highlight the value in comparing spatial cognition in progressively more unique species and environments in order to develop a more comprehensive understanding of spatial cognition across a wider range of species from more diverse niches.

## Data Availability

No new data were created or analyzed in this study.

## Abbreviations

The following abbreviations are used in this manuscript:

HF	Hippocampal formation
EC	Entorhinal cortex
LEC	Lateral entorhinal cortex
MEC	Medial entorhinal cortex
DG	Dentate gyrus
